# Factors influencing Family Medicine Residents attitudes toward mental illness

**DOI:** 10.1192/j.eurpsy.2023.1894

**Published:** 2023-07-19

**Authors:** A. Jaoua, M. H. Salhi, A. Ben Haouala, L. Gaha

**Affiliations:** Monastir tunisia, fattouma bourguiba hospital monastir, mourouj 1, Tunisia

## Abstract

**Introduction:**

Stigmatization of mental illness by health care professionals is notuncommon, and it represents a source of suffering for patients inaddition to the primary illness.

**Objectives:**

To research factors influencing family medicine residents perceptions of psychiatric pathologies.

**Methods:**

This is an analytical cross-sectional study among family medicineresidents enrolled at the Faculty of Medicine in Monastir (Tunisia),conducted over a period of 3 months (July 2022 to October 2022). TheCAMI (Community Attitudes towards the Mentally Ill) scale was usedto assess the attitude towards mental illness. Sociodemographic datawere collected through a pre-established questionnaire. The data wereanalyzed using SPSS software 26 th version. Percentage comparisonson independent series were performed using the Pearson chi-squaretest.

**Results:**

Our population was made up of 95 family medicine residents, dividedinto 28 males and 67 females. Sex ratio was 2.39. The average of agewas 28 years with extremes 25 and 35 years. 47.4% of residents(n=45) had a positive attitude towards mental illness. The associationbetween this positive attitude and a previous management of apatient with a mental illness was significant (pless than0.05). On the otherhand, there was no significant association neither with the existenceof a personal or family psychiatric history nor with the passagethrough a psychiatric internship of the residents towards mentalillness.

**Conclusions:**

The management of patients with mental illness canreduce the stigmatization of mental illness by health professionals.Measures to raise awareness and create empathetic attitudestowards the mentally ill during physician training are needed toimprove the quality of front-line care.

**Disclosure of Interest:**

None Declared

